# COPD-MMDDxNet: a multimodal deep learning framework for accurate COPD diagnosis using electronic medical records

**DOI:** 10.3389/fmed.2025.1601736

**Published:** 2025-07-11

**Authors:** Yuanyuan Yi, Lei Shi, Haoran Liu, Mingyu Wang, Min Feng, Yanxia Li

**Affiliations:** ^1^Department of Respiratory Medicine, The First Affiliated Hospital of Dalian Medical University, Dalian, China; ^2^Department of Emergency Medicine, The First Affiliated Hospital of Dalian Medical University, Dalian, China; ^3^Dalian Medical University, Dalian, China

**Keywords:** COPD, multimodal deep learning, diagnosis, cross-modal interaction, electronic medical records

## Abstract

COPD affects approximately 391 million people globally. While spirometry is recognized as the gold standard for diagnosing COPD according to the GOLD guidelines, its availability is limited in primary healthcare settings, particularly in low- and middle-income countries. Furthermore, spirometry requires patient cooperation, which may be challenging for individuals with physical limitations or comorbidities, potentially impacting its accuracy. As a result, there is a need for alternative diagnostic methods, particularly those suited for resource-constrained environments. This study proposes a novel multimodal deep learning framework, COPD-MMDDxNet, which integrates structured pulmonary CT reports, blood gas analysis, and hematological analysis from electronic medical records (EMRs) to overcome the limitations of existing diagnostic methods. This framework develops the first multimodal diagnostic tool for COPD that does not rely on spirometry. It innovatively fuses cross-modal data, incorporating four key components: parametric numerical embedding, hierarchical interaction mechanisms, contrastive regularization strategies, and dynamic fusion coefficients. These innovations significantly enhance the model's ability to capture complex cross-modal relationships, thereby improving diagnostic accuracy.The dataset used in this study comprises 800 COPD patients, with a balanced age and sex distribution, and data were collected over a 24-month period. Experimental results demonstrate that COPD-MMDDxNet outperforms traditional single-modality models and other state-of-the-art multimodal models in terms of accuracy (81.76%), precision (78.87%), recall (77.78%), and F1 score (78.32%). Ablation studies confirm the critical importance of each model component, particularly the contrastive learning module and cross-modal attention mechanism, in enhancing model performance.This framework offers a robust solution for more accurate and accessible COPD diagnosis, particularly in resource-constrained environments, without the need for spirometry.

## 1 Introduction

Chronic Obstructive Pulmonary Disease (COPD) is a serious chronic condition affecting approximately 391 million people globally (1). The primary risk factors for COPD include smoking, air pollution, and occupational exposure to harmful substances (2). Early diagnosis and management are crucial for improving patient outcomes, as timely identification of COPD plays a significant role in reducing acute exacerbations and slowing disease progression (3).

Although the Global Initiative for Chronic Obstructive Lung Disease (GOLD) guidelines recognize spirometry as the gold standard for diagnosing COPD, its availability in primary healthcare facilities is limited (4). Particularly in low- and middle-income countries, where the availability of spirometry equipment remains limited, failing to meet the screening needs of the population (5). Furthermore, spirometry requires patients to perform forced expiratory maneuvers, which may be challenging for patients with physical limitations or comorbidities (6). Therefore, despite its importance as a diagnostic tool, spirometry has inherent limitations, particularly in certain clinical and resource-constrained settings. In this context, multi-view data—owing to its rich complementary and consistent information across modalities—has garnered substantial interest for its potential to significantly enhance the performance of downstream diagnostic tasks (7).

Given these challenges, it is crucial to research disease diagnosis and treatment in resource-limited environments (8). Developing alternative diagnostic approaches that do not rely on spirometry, particularly non-invasive technologies, is essential. This study presents a solution in the form of a deep learning model specifically designed for diagnosing COPD in environments where spirometry may not be accessible.

In recent years, the rapid development of artificial intelligence (AI) technology has provided new opportunities for the diagnosis and treatment of COPD. Gompelmann et al. (9) investigate the application of AI software, ArtiQ.PFT, to assist pulmonologists in diagnosing interstitial lung disease by improving the interpretation of pulmonary function tests. Zhao et al. (10) proposed a Multi-Sentence Complementary Generative Adversarial Network to synthesize COPD-related diagnostic images by fusing semantic information from multiple clinical narrative sentences–leveraging BERT for semantic feature extraction, a multi-semantic fusion module, a pre-trained cross-modal contrast similarity model for fine-grained loss, and a multi-sentence joint discriminator to ensure that the generated images accurately reflect all input descriptions. Alves Pegoraro et al. (11) employed a Hidden Markov Model to analyze a range of breathing variables, including daily measures of oxygen therapy duration, mean breathing rate, and mean inspiratory amplitude, with the goal of detecting pre-exacerbation periods in COPD patients. However, existing studies primarily rely on single-modal data, such as pulmonary function tests or imaging, limiting the comprehensiveness of COPD diagnosis. At the same time, the widespread adoption of electronic medical record (EMR) systems globally has provided rich multimodal data. When effectively utilized, this information can enhance diagnostic accuracy.

Quennelle et al. (12) utilized EMR data to extract rare adverse events associated with pediatric cardiac catheterization, applying active learning (AL) methods to annotate the dataset, which was subsequently used to train a deep learning text classifier, incorporating clinical expert knowledge. Takeuchi et al. (13) applied natural language processing and machine learning techniques to unstructured electronic health records (EHRs) of lung cancer patients. They developed a method to predict tumor response evaluations, treatment durations, and survival curves, thereby aiding researchers and clinicians in analyzing and utilizing EHRs more efficiently.

These studies demonstrate that integrating AI with EMRs can uncover latent information within patient data, providing new approaches for early diagnosis and personalized treatment of COPD. However, current COPD diagnostic practices have yet to fully leverage the multimodal information contained in EMRs, limiting the effectiveness of AI applications in this field. Therefore, developing new methods to fully exploit the multimodal data within EMRs is crucial for improving the accuracy and efficiency of COPD diagnosis.

To address these challenges, this study proposes a novel multimodal deep learning framework, COPD-MMDDxNet, which integrates pulmonary imaging reports, medical history texts, and structured data such as blood gas analysis and hematological analysis from EMR systems to overcome the limitations of existing diagnostic methods. The proposed framework introduces four key innovations to address the challenges in COPD diagnosis:

- **Parametric numerical embedding:** A nonlinear representation mechanism utilizing learnable affine parameters replaces conventional linear transformations, enabling adaptive feature scaling across heterogeneous data modalities.
- **Hierarchical interaction mechanism:** Gated cross-attention layers facilitate multi-granular feature synergy between imaging biomarkers and physiological parameters, mitigating information degradation in shallow fusion architectures.
- **Contrastive regularization strategy:** Cross-modal semantic consistency constraints are implemented through prototype alignment in latent space, enhancing robustness against clinical data noise and missing values.
- **Dynamic fusion coefficients:** Attention-weighted fusion gates automatically calibrate hierarchical feature contributions based on pathological relevance, simultaneously optimizing prediction accuracy and model interpretability.

## 2 Method

### 2.1 Dataset construction and preprocessing

The data for this study were sourced from the electronic medical records (EMR) system of the First Affiliated Hospital of Dalian Medical University, with ethical review approval. A total of 800 COPD patients who met the inclusion criteria, based on clinical manifestations such as chronic cough, excessive sputum production, and airflow limitation, were enrolled. The cohort consisted of 276 female patients and 524 male patients, with ages ranging from 35 to 91 years, following a normal distribution. Patients with incomplete records or those who did not undergo necessary examinations were excluded. The dataset comprises multimodal medical records, collected retrospectively over a 24-month period. The raw dataset includes unstructured EMR texts and structured laboratory parameters. All personally identifiable information was anonymized using hash encryption.

For the unstructured features of the EMRs, medical natural language processing (NLP) techniques were employed for information extraction. Symptom duration, triggering factors, and other clinical elements in chief complaints were converted into standardized fields using regular expressions matched with a medical ontology database. Radiological features in pulmonary CT reports underwent entity recognition and terminology normalization.

The structured data underwent multidimensional quality control, including numerical range validation, logical relationship verification, and outlier correction. Specifically, for the systematic missingness of C-reactive protein (CRP) measurements, a clinically context-aware imputation strategy was implemented. For patients without fever symptoms and no CRP test results, a baseline value of 3.5 mg/L for CRP was assigned, based on standard clinical practice, while retaining the original testing status markers to ensure transparency and consistency in the imputation approach. For other missing values, different imputation methods were used based on the type of variable. Continuous variables were imputed using mean imputation, while categorical variables were imputed using the most frequent value.

Finally, discrete variables were encoded using binary representations, and continuous variables were Z-score standardized before model integration. The final multidimensional dataset consisted of 11 structured feature fields and 2 standardized textual description units, providing a reliable data foundation for subsequent analysis.

### 2.2 Model

**Figure 1:**
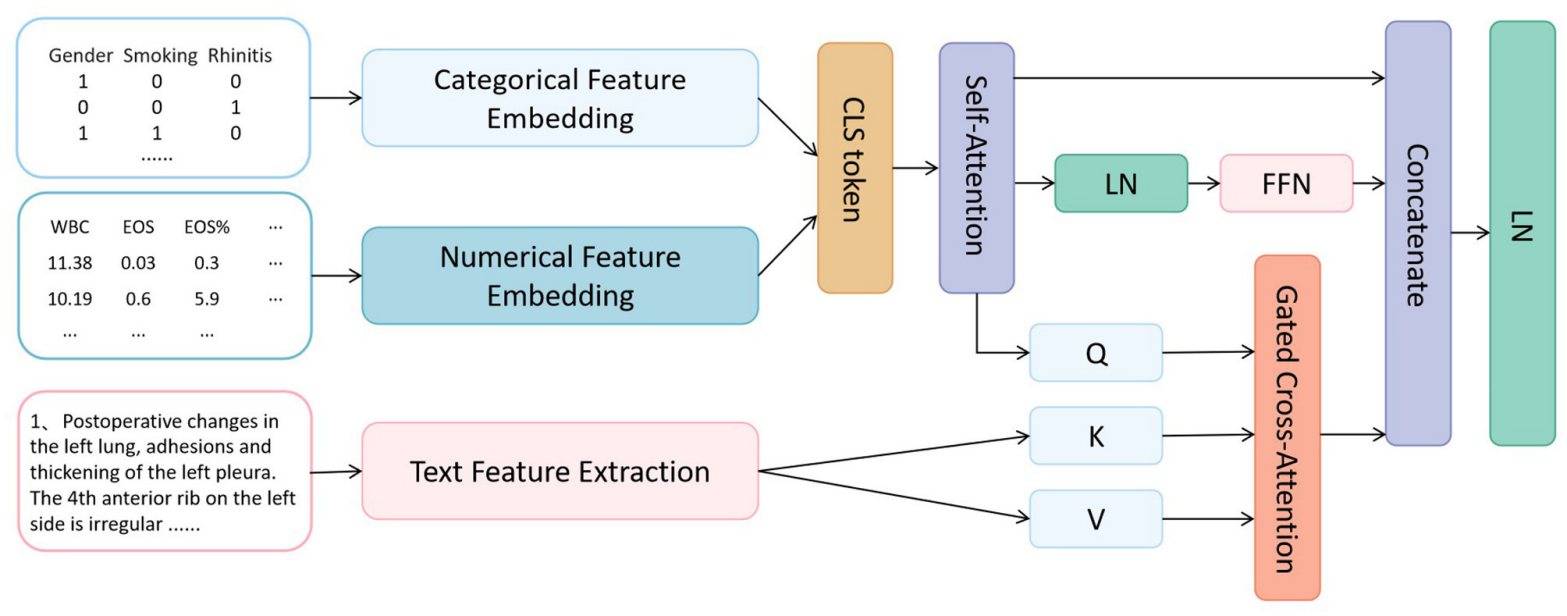
COPD-MMDxNet architecture.

The proposed COPD-MMDxNet multimodal deep learning framework, with its core mechanism centered on establishing a hierarchical progressive feature interaction process, achieves deep integration of multimodal EMR information through parametric numerical embedding, cross-modal attention routing, and contrastive regularization strategies. The model architecture is shown in Figure 1.

#### 2.2.1 Multimodal feature encoding layer

In the feature extraction stage of this study, the data from three modalities achieve in-depth semantic representation through differentiated encoding strategies.

##### 2.2.1.1 Structured feature encoding

For categorical features, a learnable embedding matrix *E*∈ℝ^|*C*| × *d*^ is adopted, where $|C|=\overset{n}{\underset{i=1}{\sum }}c_{i}$ represents the total number of categories (including special identifiers). Given the input $x_{\text{catalog}}\in {\mathbb{R}}^{k\times m}$, its embedding process is defined as:


(1) $$\begin{array}{l} {\tilde{x}}_{i}^{\text{catalog}}=E\left[x_{i}+\phi_{i}\right],\;\phi_{i}=\text{offset}\left(c_{i,j}\right) \end{array}$$

where ϕ_*i*_ is the category offset to avoid ID conflicts.

For numerical features, non - linear embedding is achieved through parameterized affine transformation:


(2) $$\begin{array}{l} {\tilde{x}}_{j}^{\text{num}}=w_{j}⊙x_{j}^{\text{num}}+b_{j},\;w_{j},b_{j}\in {\mathbb{R}}^{d} \end{array}$$

where ⊙ denotes element - wise multiplication, and *w*_*j*_, *b*_*j*_ are learnable parameters.

##### 2.2.1.2 Text feature extraction

In the text feature extraction process, we use a pre-trained BERT-Base model, which was trained on the English Wikipedia and the BookCorpus dataset (the original pre-training corpora for BERT). This model is widely used in natural language processing tasks and has demonstrated strong performance across a variety of benchmark tasks. To ensure efficient feature extraction, we project the final hidden states from BERT and normalize them to align with features from other modalities. Let *T* represent the input text and *l* the sequence length. The feature space alignment is achieved through a projection layer $P:{\mathbb{R}}^{l\times d_{\text{bert}}}\to {\mathbb{R}}^{l\times d}$:


(3) $$\begin{array}{ll} H^{\text{text}} & =\text{BERT}\left(T\right)\in {\mathbb{R}}^{l\times d_{\text{bert}}} \end{array}$$

(4) $${\hat{H}}^{\text{text}}=\text{LayerNorm}(\text{GELU}(W_{p}H^{\text{text}}+b_{p}))$$

**Cross-modal attention:** We design a bidirectional Gated Cross-Attention (GCA) mechanism to facilitate feature interaction between modalities:


(5) $$\begin{array}{ll} Q & =W_{q}\cdot \text{LN}\left(X^{\left(l\right)}\right) \end{array}$$

(6) $$K,V=W_{kv}\cdot \text{LN}({\hat{H}}^{\text{text}})$$

(7) $$\text{GCA}(X,H)\;=\text{Softmax}\left(\frac{QK^{T}}{\sqrt{d_{k}}}\right)V⊙\sigma (W_{g}[X^{\left(l\right)};{\hat{H}}^{\text{text}}])$$

where σ represents the Sigmoid gating function, and *W*_*g*_ is the learnable gating parameter that controls the cross-modal interaction.

**Feature fusion:** We adopt a residual connection and hierarchical fusion strategy to combine the features from different modalities:


(8) $$X_{\text{out}}^{\left(l\right)}=X^{\left(l\right)}+\text{FFN}(\text{LN}(X^{\left(l\right)}))+\lambda \cdot \text{GCA}(X^{\left(l\right)},{\hat{H}}^{\text{text}})$$

where λ is an adjustable fusion coefficient, allowing the dynamic adjustment of the contributions from different modalities through learnable parameters.

#### 2.2.2 Contrastive regularization and dynamic classifier

To enhance cross-modal semantic consistency, we introduce a contrastive loss based on InfoNCE: **Feature projection:** We establish a shared projection space $Z\in {\mathbb{R}}^{d_{z}}$ to project features from different modalities into a common space:


(9) $$\begin{array}{ll} z^{\text{struct}} & ={\text{Proj}}_{\theta }\left(x_{\text{cls}}\right) \end{array}$$

(10) $$\begin{array}{ll} z^{\text{text}} & ={\text{Proj}}_{\theta }\left(h_{\text{cls}}^{\text{text}}\right) \end{array}$$

where Proj_θ_ denotes the projection function with learnable parameters θ, mapping features from the structured data (*x*_cls_) and text ($h_{\text{cls}}^{\text{text}}$) into the shared feature space Z.

**Loss function:** We define the symmetric contrastive loss as follows:


(11) $$\begin{array}{l} L_{\text{cont}}=-\frac{1}{2N}\overset{N}{\underset{i=1}{\sum }}\left[\log\frac{e^{s\left(z_{i}^{\text{struct}},z_{i}^{\text{text}}\right)/\tau }}{\sum_{j=1}^{N}e^{s\left(z_{i}^{\text{struct}},z_{j}^{\text{text}}\right)/\tau }}+\log\frac{e^{s\left(z_{i}^{\text{text}},z_{i}^{\text{struct}}\right)/\tau }}{\sum_{j=1}^{N}e^{s\left(z_{i}^{\text{text}},z_{j}^{\text{struct}}\right)/\tau }}\right] \end{array}$$

where *s*(·) represents the cosine similarity function, and τ is a learnable temperature parameter that controls the scale of the similarity between different feature pairs.

#### 2.2.3 Joint optimization objective

The final objective function for multi-task joint optimization is given by:


(12) $$\begin{array}{l} L_{\text{total}}=\alpha \cdot L_{\text{task}}+\beta \cdot L_{\text{cont}}+\gamma \cdot L_{\text{reg}} \end{array}$$

where $L_{\text{task}}$ represents the main task loss (such as cross-entropy), $L_{\text{reg}}$ is the weight regularization term, and α, β, and γ are the balance coefficients that control the contribution of each loss term.

## 3 Experiments

This section presents experiments designed to evaluate the effectiveness of the COPD-MMDxNet framework. Initially, we assess single-modality models to underscore the limitations inherent in relying on individual data sources. Subsequently, we compare our multimodal approach against other state-of-the-art methods to demonstrate its relative advantages. Finally, ablation studies are conducted to investigate the impact of critical components, including the contrastive learning module and the cross-modal attention mechanism. These experiments collectively highlight the superior performance of our model and emphasize the significance of each component in enhancing diagnostic accuracy and robustness.

### 3.1 Experiment setup and computational costs

In all experiments, we employed a 5-fold cross-validation approach for model training and evaluation. This method ensures robust performance assessment by splitting the dataset into five subsets, with each subset serving as the validation set once while the remaining four are used for training. The results from each fold were averaged to obtain the final performance metrics, mitigating the risk of overfitting and providing a comprehensive evaluation of the model's generalization ability.

For computational efficiency, all training was conducted on a single NVIDIA V100 GPU. Each fold of the 5-fold cross-validation took approximately 10 min, with the total training time for the entire process being 50 min on the V100 GPU. To optimize memory usage, we implemented dynamic batching and gradient accumulation during training.

### 3.2 Comparison with single-modality baseline models

To validate the effectiveness of multimodal diagnosis, this study first performed a comparative experiment with single-modality baseline models. The single-modality models were based on structured clinical data and textual data analysis, respectively. The experimental results are shown in Table 1.

**Table 1:** Comparison of single-modality baseline models.

**Data**	**Method**	**Acc**	**Prec**	**Rec**	**Spec**	**F1 score**
CSV	XGBoost	0.6706	0.6909	0.4935	0.8172	0.5758
CSV	FT-Transformer	0.6433	0.5652	0.3881	0.8077	0.4602
TEXT	DPCNN	0.6941	0.6769	0.5867	0.7789	0.6286
TEXT	BERT	0.7176	0.7077	0.6133	0.8000	0.6571
**CSV&TEXT**	**COPD-MMDxNet**	**0.8176**	**0.7887**	**0.7778**	**0.8469**	**0.7832**

The bolding indicates the parts where our model stands out.

The experimental results indicate that while the traditional XGBoost (14) model and FT-Transformer (15) provide certain predictive capabilities using pulmonary function parameters and inflammatory biomarkers, they exhibit high missed diagnosis rates and low recall when handling early-stage cases, highlighting the limitations of single-modality data in capturing complex pathological mechanisms. In textual data analysis, although the DPCNN (16) and BERT (17) models can extract useful information from radiological reports, and BERT improves accuracy, it still struggles to effectively capture dynamic imaging features and exhibits relatively low specificity. This reflects the shortcomings of single-modality text models in integrating and analyzing diverse features. The COPD-MMDxNet framework, by integrating multimodal information from both structured data and text, successfully overcomes the limitations of single-modality models in capturing cross-dimensional pathological relationships. This demonstrates that multimodal fusion can effectively reduce the information omissions and biases inherent in single data sources, providing a more comprehensive representation of the complex characteristics of the disease.

### 3.3 Comparison with advanced multimodal models

To evaluate the effectiveness of the proposed COPD-MMDxNet model, we conducted comparative experiments with three representative models: RETAIN (18), BRLTM (19), and ExBEHRT (20). The experimental results are shown in Table 2. The RETAIN model is based on a reverse-time attention mechanism, which improves accuracy and interpretability by processing EHRs data in reverse time order and applying a two-level attention mechanism. BRLTM, on the other hand, uses a Transformer architecture and self-attention mechanism to capture forward and backward dependencies in sequential data, enhancing the model's temporal learning capability. ExBEHRT is an extension of BEHRT, improving performance through stacking and pre-training strategies. The experimental results are shown in the Table 2.

**Table 2:** Comparison with advanced multimodal models.

**Version**	**Acc**	**Prec**	**Rec**	**Spec**	**F1 score**
RETAIN	0.7423	0.7198	0.7045	0.7812	0.7098
BRLTM	0.7834	0.7556	0.7412	0.8145	0.7483
ExBEHRT	0.7956	0.7645	0.7534	0.8212	0.7589
**Ours**	**0.8176**	**0.7887**	**0.7778**	**0.8469**	**0.7832**

The bolding indicates the parts where our model stands out.

Compared to the three baseline models, COPD-MMDxNet demonstrates significant advantages in multiple aspects, particularly in the fusion of multimodal features and cross-modal interaction for complex EHRs data. Experimental results show that our model outperforms RETAIN, BRLTM, and ExBEHRT in terms of accuracy, precision, recall, and AUC. COPD-MMDxNet effectively integrates information from structured data, text, and numerical data through a hierarchical cross-modal interaction mechanism. This stepwise feature interaction approach allows for deeper and more accurate cross-modal information complementarity and integration, which is superior to traditional methods like unimodal self-attention and simple cross-modal mapping. Additionally, COPD-MMDxNet adopts a dynamic feature fusion strategy that adjusts the fusion weights based on the contribution of each modality, thereby enhancing the model's performance across different tasks. In contrast, RETAIN and BRLTM use fixed-weight fusion strategies, and while ExBEHRT incorporates multimodal data, it lacks an effective cross-modal consistency training mechanism, limiting the full potential of multimodal information. Furthermore, by introducing InfoNCE-based contrastive regularization, we further enhance the semantic consistency across multimodal data. These innovations enable COPD-MMDxNet to exhibit stronger capabilities in multimodal data integration, semantic consistency, and feature processing, further demonstrating its superiority in EHRs data processing.

### 3.4 Ablation analysis of key model components

We also conducted systematic ablation studies to evaluate the effectiveness of our model. The experimental results are presented in Table 3. These experiments highlight the synergistic interactions between the key components within the multimodal framework and their significant influence on the model's robustness. The results indicate that the removal of the contrastive learning module significantly reduces the alignment of cross-modal semantics, which in turn leads to an increase in false-positive cases. This can be attributed to the weakened latent correlations between modalities, impairing the model's ability to establish distinct decision boundaries in the feature space. Further analysis shows that removing the cross-modal attention mechanism severely hinders the integration of heterogeneous features. The absence of dynamic weight allocation prevents the model from effectively capturing the complex pathological correlations across modalities, which results in a substantial increase in false-negative rates.

**Table 3:** Ablation analysis of key model components.

**Version**	**Acc**	**Prec**	**Rec**	**Spec**	**F1 score**
**Original model**	**0.8176**	**0.7887**	**0.7778**	**0.8469**	**0.7832**
Remove contrastive learning	0.8000	0.7639	0.7639	0.8265	0.7639
Remove cross-modal attention	0.7895	0.7049	0.7049	0.8364	0.7049
XGBoost+BERT direct fusion	0.7647	0.7864	0.6623	0.8495	0.7183
FTTransform+BERT fusion	0.7412	0.7656	0.6282	0.8370	0.6908

The bolding indicates the parts where our model stands out.

In addition, we performed a comparison using a direct fusion approach, where FT-Transformer or XGBoost was used to process structured data features, and these features were directly concatenated with the text features processed by BERT. The experimental results show that, although this direct fusion method can extract certain structured and textual features, its performance is significantly lower than that of the proposed multimodal framework. The direct concatenation approach fails to capture the deep inter-modal correlations, preventing the model from effectively utilizing the complementary information between modalities.

Through these comparative experiments, we demonstrate the significant limitations of single-data fusion methods and cross-modal fusion strategies that lack dynamic weighting. Our framework, by leveraging more complex feature interactions and dynamic weight adjustments, is better able to capture pathological correlations between modalities, thus significantly enhancing the model's diagnostic ability and robustness.

## 4 Conclusion

This study presents COPD-MMDDxNet, a novel multimodal deep learning framework designed to enhance COPD diagnosis by integrating structured clinical data, radiological reports, and blood gas analysis from electronic medical records (EMR). The framework addresses the limitations of spirometry, making it particularly suitable for resource-constrained environments. Key innovations, including parametric numerical embedding, hierarchical interaction mechanisms, contrastive regularization, and dynamic fusion coefficients, enable effective multimodal integration and capture of complex cross-modal relationships.

Experimental results demonstrate that COPD-MMDDxNet outperforms traditional single-modality models and other state-of-the-art multimodal models in terms of accuracy, precision, recall, and F1 score. Ablation studies confirm the importance of each component, particularly the contrastive learning module and cross-modal attention mechanism, in enhancing model performance.

COPD-MMDDxNet offers a robust solution for more accurate and accessible COPD diagnosis, particularly in settings where spirometry is unavailable or impractical. This framework has the potential to improve early diagnosis and disease management in COPD. However, it is important to note that the current study was conducted using a single-center dataset, which limits the generalizability of the results. Future work will focus on expanding clinical applicability and validating the model across multiple data centers to further assess its robustness and reliability in diverse clinical environments.

The code for the model, including the architecture and training procedures, has been made publicly available on GitHub for transparency and reproducibility. The repository can be accessed at: https://github.com/yiyuanyuan827/COPD-MMDDxNet.

## Data Availability

The raw data supporting the conclusions of this article will be made available by the authors, without undue reservation.

## References

[1] Adeloye D Song P Zhu Y Campbell H Sheikh A Rudan I. Global, regional, and national prevalence of, and risk factors for, chronic obstructive pulmonary disease (COPD) in 2019: a systematic review and modelling analysis. Lancet Respir Med. (2022) 10:447–58. 10.1016/S2213-2600(21)00511-7 35279265 PMC9050565

[2] Zhao L Wang X Liu Z Wang Z Chen Z. Learnable graph guided deep multi-view representation learning via information bottleneck. IEEE Trans Circ Syst Video Technol. (2024) 35:3303–3314. 10.1109/TCSVT.2024.3509892

[3] Sun Y Zhou J. New insights into early intervention of chronic obstructive pulmonary disease with mild airflow limitation. Int J Chron Obstruct Pulmon Dis. (2019) 14:1119–1125. 10.2147/COPD.S205382 31213792 PMC6536809

[4] Agustí A Celli BR Criner GJ Halpin D Anzueto A Barnes P. Global initiative for chronic obstructive lung disease 2023 report: GOLD executive summary. J Pan African Thoracic Soc. (2022) 4:58–80.

[5] Siddharthan T Grealis K Robertson NM Lu M Liu S Pollard SL. Assessing the prevalence and impact of preserved ratio impaired spirometry in low-income and middle-income countries: a post-hoc cross-sectional analysis. Lancet Global Health. (2024) 12:e1498–505. 10.1016/S2214-109X(24)00233-X 39151984 PMC12404211

[6] Clark MV. Asthma: A Clinician's Guide. Burlington: Jones & Bartlett Learning. (2010).

[7] Zhao L Xie Q Li Z Wu S Yang Y. Dynamic graph guided progressive partial view-aligned clustering. IEEE Trans Neural Netw Learn Syst. (2024) 36:9370–9382. 10.1109/TNNLS.2024.3425457 39042549

[8] Wang S Zheng Z Wang X Zhang Q Liu Z. A cloud-edge collaboration framework for cancer survival prediction to develop medical consumer electronic devices. IEEE Trans Consum Electr. (2024) 70:5251–5258. 10.1109/TCE.2024.3413732

[9] Gompelmann D Gysan MR Desbordes P Maes J Van Orshoven K De Vos M. AI-powered evaluation of lung function for diagnosis of interstitial lung disease. Thorax. (2025) 13:221537. 10.1136/thorax-2024-221537 40081903 PMC12322453

[10] Zhao L Huang P Chen T Fu C Hu Q Zhang Y. Multi-sentence complementarily generation for text-to-image synthesis. IEEE Trans Multim. (2023) 26:8323–8332. 10.1109/TMM.2023.3297769

[11] Alves Pegoraro J Guerder A Similowski T Salamitou P Gonzalez-Bermejo J Birmelé E. Detection of COPD exacerbations with continuous monitoring of breathing rate and inspiratory amplitude under oxygen therapy. BMC Med Inform Dec Making. (2025) 25:101. 10.1186/s12911-025-02939-3 40001140 PMC11863910

[12] Quennelle S Malekzadeh-Milani S Garcelon N Faour H Burgun A Faviez C. Active learning for extracting rare adverse events from electronic health records: a study in pediatric cardiology. Int J Med Inform. (2025) 195:105761. 10.1016/j.ijmedinf.2024.105761 39689449

[13] Takeuchi T Horinouchi H Takasawa K Mukai M Masuda K Shinno Y. A series of natural language processing for predicting tumor response evaluation and survival curve from electronic health records. BMC Med Inform Decis Mak. (2025) 25:1–11. 10.1186/s12911-025-02928-6 39962486 PMC11834625

[14] Chen T Guestrin C. Xgboost: a scalable tree boosting system. In: Proceedings of the 22nd ACM SIGKDD International Conference on Knowledge Discovery and Data Mining (2016). p. 785–794. 10.1145/2939672.2939785

[15] Gorishniy Y Rubachev I Khrulkov V Babenko A. Revisiting deep learning models for tabular data. In: Advances in Neural Information Processing Systems (2021). p. 18932–43.

[16] Zhang M Pang J Cai J Huo Y Yang C Xiong H. DPCNN-based models for text classification. In: 2023 IEEE 10th International Conference on Cyber Security and Cloud Computing (CSCloud)/2023 IEEE 9th International Conference on Edge Computing and Scalable Cloud (EdgeCom). IEEE (2023). p. 363–368. 10.1109/CSCloud-EdgeCom58631.2023.00068

[17] Devlin J Chang MW Lee K Toutanova K. Bert: pre-training of deep bidirectional transformers for language understanding. In: Proceedings of the 2019 conference of the North American chapter of the association for computational linguistics: human language technologies, volume 1 (long and short papers) (2019). p. 4171–4186.

[18] Choi E Bahadori MT Sun J Kulas J Schuetz A Stewart W. Retain: an interpretable predictive model for healthcare using reverse time attention mechanism. In: Advances in Neural Information Processing Systems. (2016). p. 29.

[19] Meng Y Speier W Ong MK Arnold CW. Bidirectional representation learning from transformers using multimodal electronic health record data to predict depression. IEEE J Biomed Health Inform. (2021) 25:3121–9. 10.1109/JBHI.2021.3063721 33661740 PMC8606118

[20] Rupp M Peter O Pattipaka T. Exbehrt: extended transformer for electronic health records. In: International Workshop on Trustworthy Machine Learning for Healthcare. Springer (2023). p. 73–84. 10.1007/978-3-031-39539-0_7

